# Gut microbiota profiles as potential indicators of irradiation-induced quality loss in mass-reared *Aedes albopictus* males

**DOI:** 10.20517/mrr.2026.25

**Published:** 2026-08-28

**Authors:** Paerhande Dilinuer, Jehangir Khan, Ming Li, Yu Wu, Xiaoying Zheng, Zhongdao Wu, Datao Lin, Hanano Yamada, Jeremy Bouyer, Kostas Bourtzis, Dongjing Zhang

**Affiliations:** ^1^Chinese Atomic Energy Agency Center of Excellence on Nuclear Technology Applications for Insect Control, Key Laboratory of Tropical Disease Control of the Ministry of Education, Sun Yat-sen University, Guangzhou 510080, Guangdong, China.; ^2^International Atomic Energy Agency Collaborating Centre, Sun Yat-sen University, Guangzhou 510080, Guangdong, China.; ^3^Guangdong Provincial Engineering Technology Research Center for Diseases-Vectors Control, Sun Yat-sen University, Guangzhou 510080, Guangdong, China.; ^4^Zoology Department, Abdul Wali Khan University Mardan, Khyber Pakhtunkhwa 23200, Pakistan.; ^5^Insect Pest Control Section, Joint FAO/IAEA Centre of Nuclear Techniques in Food and Agriculture, Department of Nuclear Sciences and Applications, International Atomic Energy Agency, Vienna 1400, Austria.; ^6^ASTRE, Cirad, INRAE, Université de Montpellier, Plateforme Technologique CYROI, Sainte-Clotilde, La Réunion 97490, France.

**Keywords:** Sterile insect technique, cultivable gut-associated bacteria, 16S *rRNA*, quality control, microbial community composition

## Abstract

**Aim:** The Sterile Insect Technique is a promising method for *Aedes albopictus* control, but its effectiveness hinges on male quality, which may be compromised during mass-rearing and irradiation. Reliable biological indicators capable of reflecting irradiation-induced quality loss remain limited. Gut microbiota regulates mosquito development, immunity, and metabolism and may therefore provide potential indicators of sterile male quality. This study explored whether gut microbiota composition can reflect irradiation-induced quality impairment in males of a long-term (over 3 years) mass-reared *Ae. albopictus* GT strain.

**Methods:** Male pupae were exposed to X-ray irradiation without filtration at an estimated absorbed dose of 78 Gy. Emergence rate, flight ability, longevity, metabolic capacity, and gut microbiota (via 16S *rRNA* gene sequencing, culture-based methods, and qPCR) were compared between irradiated and non-irradiated males.

**Results:** Irradiation significantly reduced emergence rate, flight ability, and longevity. It did not alter gut microbiota diversity, but reshaped its composition, including the emergence of *Sphingobacterium*, increased abundance of *Stenotrophomonas* and *Comamonas*, and decreased levels of *Delftia* and *Achromobacter*. Similarity was observed in the culturable gut bacterial community, confirmed by qPCR with eight selected key taxa. Metabolic changes included reduced glutamine synthetase activity and elevated glucose levels in irradiated males.

**Conclusion:** These results show that irradiation-induced quality loss is accompanied by reproducible shifts in gut microbiota composition and metabolic status. These findings suggest that gut microbiota profiles may serve as potential indicators of irradiation-associated quality changes in sterile males and provide preliminary evidence for microbiota-informed quality assessment approaches in *Ae. albopictus* SIT programs.

## INTRODUCTION

*Aedes albopictus* is a highly invasive species responsible for the transmission of several major mosquito-borne diseases, including dengue, chikungunya, and Zika^[1-4]^. In the absence of effective vaccines or antiviral treatments, vector control remains the primary disease management strategy, with the Sterile Insect Technique (SIT) gaining increasing attention as a species-specific and environmentally friendly strategy for suppressing mosquito populations^[5,6]^.

SIT involves the release of mass-reared, radiation-sterilized male mosquitoes into the field, where they compete with wild males to mate with wild females, resulting in infertile matings and subsequent population suppression^[5]^. Its success critically depends on the quality and competitiveness of the released males, yet key SIT processes including mass-rearing, irradiation, handling, transportation and release may impair critical quality traits (e.g., flight ability, longevity, and mating competitiveness) of sterile males, undermining the effectiveness of SIT programs^[5,7]^. Identifying sensitive biological indicators for such stress-induced quality loss remains a major challenge for SIT quality control.

Gut-associated microbiota have a pivotal role in mosquito biology, regulating digestion, immune responses, development and vector competence^[8-11]^, making them promising quality indicators. Mosquito larvae acquire gut microbes mainly from aquatic environments^[12,13]^, with environmental factors, such as fertilizer-mediated changes in aquatic microbial communities, influencing larval development^[14]^. As holometabolous insects, mosquitoes undergo substantial shifts in gut microbiota composition across different life stages. Additionally, there are marked differences between male and female mosquitoes^[13,15]^. Sex-specific differences in gut microbiota have been observed, with female mosquitoes, which feed on vertebrate blood to acquire essential proteins and iron for egg production, harboring distinct gut microbial profiles compared to males, which primarily consume sugar-rich diets with a lower pH^[10,16]^. These findings highlight the complex interplay between environmental inputs, host physiology, and microbial communities.

Irradiation is known to alter gut microbial diversity and composition in insects, with such shifts reflecting host physiological status and stress intensity. For example, in *Bactrocera dorsalis*, irradiation reduced bacterial richness and shifted the relative abundance of key taxa^[17]^. Similarly, in *Ae. albopictus*, irradiation increased the relative abundance of *Aeromonas* and *Elizabethkingia* species^[18]^. These irradiation-induced microbial shifts may reflect physiological stress and could adversely affect sterile male performance, including survival and mating behavior^[17,19,20]^. Despite these insights, the interaction between irradiation and gut microbiota has not been systematically explored in mass-reared *Ae. albopictus* males.

This study investigated whether gut microbiota composition is associated with irradiation-induced quality loss in mass-reared *Ae. albopictus* males, using a *Wolbachia*-free *Ae. albopictus* GT strain maintained under mass-rearing conditions since 2020^[19]^. Male pupae were irradiated by X-ray without filtration at an estimated absorbed dose of 78 Gy [95% confidence interval (CI): 74.1-81.9 Gy]. Core quality parameters, including emergence rate, flight ability, and adult longevity, were evaluated in parallel with gut microbiota profiling across developmental stages using culture-dependent methods, 16S *rRNA* gene sequencing, and targeted quantitative real-time PCR (qPCR) validation. Metabolic parameters were further analyzed to explore whether microbiota shifts co-occur with physiological changes relevant to sterile male performance. Through this integrated approach, we aimed to evaluate the potential of gut microbiota profiles as indicators of sterile male quality in SIT programs.

## METHODS

### Mosquito mass rearing and irradiation treatment

The mosquito strain used in this study was the *Ae. albopictus* GT strain, derived from the wild-type GUA strain through tetracycline treatment to eliminate *Wolbachia* infection^[19]^. This strain has been mass-reared at the mosquito facility of Sun Yat-sen University (SYSU) since December 2020. The egg collection, hatching, larval rearing, adult rearing and blood-feeding procedures were the same as those described in a previous study^[19]^. Adult females were blood-fed using commercially obtained sterile defibrinated sheep blood (CHINOOK, Ararat Biotechnology Co., Guangzhou, China). Rearing conditions were maintained at 27 ± 2 °C, with a 12:12 h light:dark (L:D) photoperiod and 75% ± 10% relative humidity (RH).

Male pupae (24-36-hour-old) were transported from the mosquito facility to the North Campus of SYSU in a refrigerated vehicle within 2 h. The pupae were irradiated using a RadSource 2000 Pro X-ray irradiator (160 kV, 25 mA) at an absorbed dose of 78 Gy (95% CI: 74.1-81.9 Gy). The irradiation was performed without copper filtration. This absorbed dose exceeds the fully sterilizing dose reported for *Ae. albopictus* males (30 Gy in our conditions). After irradiation, male pupae were allowed to emerge, and adults were maintained on a 10% glucose solution sterilized by autoclaving (121 °C, 20 min). Non-irradiated males served as controls. All mosquitoes were maintained at 28 ± 1 °C, 70% ± 10% RH, under a 12:12 (L:D) cycle. Over 99.0% sterility was induced between irradiated males and normal females under such exposure conditions as previously reported^[21]^.

### Mosquito quality assessment

To evaluate the effect of irradiation on adult emergence, 30 irradiated or control pupae were placed in small plastic cups inside a rearing cage. After 48 h, emerged adults (fully escaped from the exuviae) were counted. Individuals that failed to emerge or died during emergence were considered as non-viable. Six replicates were performed for each group.

To assess flight ability, 100 anesthetized male mosquitoes from each group were randomly selected and transferred to a sterile Petri dish. Mosquitoes were anesthetized by placing each group in a 4 °C cold chamber; within 30 min, individuals were gently transferred into Petri dishes using a fine brush. After removal from the cold chamber, mosquitoes were maintained at room temperature (the flight assay room) for 2 h to allow full recovery prior to testing. A transparent cylindrical flight chamber (10 cm diameter, 80 cm height, 2 mm wall thickness) was placed over the Petri dish, and mosquitoes were allowed to escape for 6 minutes at room temperature. Further details of the flight ability assay are provided in supplementary materials [Supplementary Figure 1]. Flight ability (%) was calculated as the percentage of mosquitoes that successfully escaped from the flight chamber: Flight ability (%) = [100 - (m + n)] / 100 × 100%, where m and n represent the numbers of mosquitoes remaining in the dish and on the chamber wall, respectively. Eighteen replicates were performed per group.

To measure male longevity, 30 irradiated or control males were initially placed in separate cages, with three replicate cages per treatment group (90 males per group). Mortality was recorded daily, and dead mosquitoes were removed from the cages during each observation. During routine daily handling, a small number of live mosquitoes were inadvertently lost from the cages. Because the exact time of loss could not be determined retrospectively, these individuals were not assigned artificial death or censoring times. The numbers of mosquitoes with traceable outcomes were therefore slightly lower than the initial sample size. Two feeding regimes were used: (1) 10% glucose solution (standard condition) and (2) sterilized distilled water (stress condition), with three replicates per group. To account for the potential non-independence of mosquitoes within the same cage, an additional cage-level sensitivity analysis was performed using replicate cages as the experimental unit.

### 16S rRNA sequencing analysis

16S *rRNA* gene sequencing was used to compare the gut microbiota between irradiated and control males. Samples were collected from 40 individuals, including pupae and 1-, 4-, and 7-day-old adults, for both irradiated and non-irradiated treatments. Each group consisted of five biological replicates, with each replicate comprising 20 dissected guts. Adults were collected using an aspirator, anesthetized with CO_2_, and maintained on a 10% sugar solution. After collection, the mosquitoes were surface-disinfected by dipping in 70% ethanol (1 min), rinsed in sterile 1 × phosphate-buffered saline (PBS), and stored at -80°C prior to DNA extraction. Gut dissections were performed using sterilized instruments under aseptic conditions to minimize potential contamination during sample preparation. Sterile consumables were used throughout sample processing.

DNA was extracted using the QIAamp Fast DNA Stool Mini Kit (Qiagen, USA) and purified with the DNA Gel Extraction Kit (Axygen, USA) according to the manufacturer’s protocols. The V3-V4 regions of the 16S *rRNA* gene were amplified using primers 338F (ACTCCTACGGGAGGCAGCA) and 806R (GGACTACHVGGGTWTCTAAT). Amplicons were pooled in equimolar concentrations and paired-end sequenced on an Illumina NextSeq 2000 platform (Illumina, San Diego, USA) by Majorbio Bio-Pharm Technology Co. Ltd. (Shanghai, China).

Raw FASTQ files were demultiplexed using a custom Perl script, quality-filtered with fastp (version 0.19.6), and merged using FLASH (version 1.2.7). These analyses were performed via the above-mentioned platform of Majorbio. Details on quality filtering were as follows: (i) reads truncated when the average quality score dropped below Q20 over a 50 bp sliding window; (ii) only overlapping sequences longer than 10 bp were assembled based on their overlapping region, with a maximum mismatch ratio of 0.2; (iii) samples were distinguished based on barcodes and primers, with exact barcode matching and a 2-nucleotide mismatch allowed in primer matching. The optimized sequences were clustered into operational taxonomic units (OTUs) at a 97% sequence similarity using UPARSE version 7.1. Taxonomic classification of each OTU representative sequence was performed using the RDP Classifier (version 2.2) against the Silva v138 database at a 0.7 confidence threshold. Functional predictions were made using PICRUSt2 (Phylogenetic Investigation of Communities by Reconstruction of Unobserved States)^[22]^.

### Cultivable gut-associated bacterial species

To evaluate irradiation effects on the cultivable gut-associated bacterial species, the entire gut (from foregut to hindgut) was dissected and collected from males at 4 stages: pupae (24-36 h post-pupae) and adults at 1, 4, and 7 days post-emergence. Three replicates were performed for each age group, with 20 guts per replicate. Samples were homogenized and plated on selective media: Columbia blood agar (CBA), nutrient broth agar (NA), LB agar (LB), tryptic soy broth agar (TSA), and brain-heart infusion broth agar (BHI). Bacterial colonies were identified by MALDI-TOF MS (Matrix-Assisted Laser Desorption/Ionization Time-of-Flight Mass Spectrometry). For species that could not be identified by MALDI-TOF MS, sequencing was performed using universal primers: 27F (AGAGTTTGATCMTGGCTCAG), 1492R (GGTTACCTTGTTACGACTT), 909F (ACTCAAAKGAATWGACGG), and 1391R (GACGGGCGGTGWGTRCA).

DNA was extracted from the cultured bacterial colonies using the FastPure Cell/Tissue DNA Isolation Mini Kit (Vazyme, China). Concentrations were measured using a NanoDrop 3000 spectrophotometer and standardized to > 15 ng/μL. PCR reactions (25 μL) included: 12.5 μL of 2×Rapid Taq Master Mix, 1 μL of primers (10 μmol/L), and 1 μL of DNA template. Thermocycling: 95 °C for 3 min, followed by 30 cycles of 15 s at 95 °C, 15 s at 55/52 °C, 5 s at 72 °C, and a final extension at 72 °C for 5 min. PCR products were visualized on a 1% agarose gel stained with 1 μg/mL ethidium bromide.

### qPCR validation of differential bacterial taxa

qPCR was performed to validate the relative abundance of eight gut bacterial taxa that were both significantly altered in the 16S *rRNA* sequencing analysis and successfully isolated through culture-based methods. These taxa included eight genera: *Delftia*, *Achromobacter*, *Stenotrophomonas*, *Leucobacter*, *Pseudomonas*, *Deinococcus*, *Comamonas*, and *Sphingobacterium*.

Total DNA was extracted from dissected gut samples using a DNA extraction Kit (Vazyme Biotech Co., Ltd., Nanjing, China; Cat. No. DC102) according to the manufacturer’s instructions. The *Ae. albopictus* ribosomal protein S6 (*rpS6*) gene served as the internal reference. Primer sequences for the eight bacterial taxa are presented in Supplementary Table 1.

qPCR amplification was performed using SYBR Green Supermix (Vazyme, Nanjing, China) on a LightCycler 96 real-time PCR system (Roche, Basel, Switzerland). Each 20 μL reaction contained 10 μL of 2×Supermix, 2 μL of DNA template, 0.4 μL of each primer (10 μmol/L), and 7.2 μL of nuclease-free water. The thermocycling protocol consisted of an initial denaturation at 95 °C for 3 min, followed by 40 cycles of 95 °C for 15 s, primer-specific annealing temperature for 30 s, and 72 °C for 30 s. Fluorescence signals were collected during the extension stage. A melting curve (65-95 °C) was generated at the end of each run to verify amplification specificity. Relative bacterial abundance was calculated using the 2^-∆Ct^ method. ∆Ct was calculated as the difference between the Cq values of the target bacterial taxon and the internal reference gene, and relative abundance was expressed directly as 2^-ΔCt^ for each individual sample.

### Assessment of metabolic activity

Based on microbial function predictions from 16S *rRNA* data, five metabolic indicators in mosquitoes were assessed to evaluate irradiation-induced physiological changes, including glutamine synthetase (GS) activity, glucose content, malondialdehyde (MDA) (a marker of lipid peroxidation), protein carbonyl content, and hydrogen peroxide levels. The rationale for selecting these indicators is as follows: glutamine synthetase activity was included because functional prediction suggested differences in glutamine metabolism between irradiated and control groups; glucose content was measured as a key indicator of carbohydrate metabolism and energy supply^[23]^; protein carbonyl and MDA were determined as classical markers of oxidative damage to proteins and lipids, respectively^[24,25]^; and hydrogen peroxide levels were assessed because previous studies have shown that irradiation can significantly affect reactive oxygen species (ROS) metabolism in insects^[26]^. The following kits were used for the metabolic indicator measurement: GS activity (Bioleaper, BSY-BR5000167-100T), MDA (CheKine**^TM^** Micro, Abbkine, KTB1050-96T), glucose content (CheKine**^TM^** Micro, KTB1300-96T), protein carbonyl content (CheKine**^TM^** Micro, Abbkine, KTB1200-96T), and hydrogen peroxide content (CheKine**^TM^** Micro, Abbkine, KTB1041). Measurements were made using a microplate reader (Agilent Technologies, SH1MF-SN) at the wavelengths specified in each protocol.

### Statistical analysis

Bioinformatics analyses of 16S *rRNA* gene sequencing data were performed using the Majorbio Cloud platform (https://cloud.majorbio.com). OTU-level alpha diversity indices (observed OTUs, Chao1, Shannon index, and Good’s coverage) were computed using Mothur v1.30.1. Beta diversity was assessed via principal coordinate analysis (PCoA) based on weighted UniFrac distance and non-metric multidimensional scaling (NMDS) using Bray-Curtis dissimilarity (Vegan v2.5-3). Group differences were tested with the permutational multivariate analysis of variance (PERMANOVA) test (Vegan v2.5-3). The linear discriminant analysis (LDA) effect size (LEfSe) (http://huttenhower.sph.harvard.edu/LEfSe) was used to identify the differentially abundant taxa (LDA score > 2, *P* < 0.05, representing statistically significant difference) across groups^[27]^. Additional statistical analysis and data visualizations were performed in GraphPad Prism v7.0 and R v3.6.3. Student’s *t*-test or Wilcoxon rank-sum test was used for two-group comparisons. One-way analysis of variance (ANOVA) with Tukey’s post-hoc test was used for multi-group comparisons. Emergence rate and flight ability were analyzed using generalized linear mixed models (GLMMs) with binomial error distribution, with treatment as a fixed effect and replicate as a random effect. Survival curves were generated using the Kaplan-Meier method and compared with the log-rank test. As a sensitivity analysis, replicate cages were treated as the experimental unit, and cage-level restricted mean survival time (RMST) was compared between treatment groups. The two-tailed Mann-Whitney test was used to compare the relative abundance of eight gut bacterial taxa between irradiated and control groups. Metabolic indicator values between irradiated and control groups were analyzed using the Mann-Whitney test. A significance threshold of *P* < 0.05 was considered statistically significant.

## RESULTS

### Irradiation reduces emergence rate, flight ability, and longevity in males

Irradiation significantly reduced both adult emergence rate and flight ability (GLMMs, *P* < 0.001 for both comparisons). The emergence rate of irradiated pupae was 78.33% ± 4.0%, significantly lower than 96.67% ± 3.0% in control groups [Figure 1A]. Flight ability was also markedly reduced in irradiated males (35.17% ± 19.32%) compared to controls (85.17% ± 6.89%) [Figure 1B]. Under 10% sugar solution feeding, irradiated males had reduced lifespans compared to control groups (Figure 1C, Log-rank test, *χ²* = 171.2, *P* < 0.0001). Notably, while 45.56% of control males survived until the 30th day, irradiated males reached 0% survival by the 23^rd^ day. Under water-only conditions, survival also differed significantly between irradiated and control males (Figure 1D, *χ²* = 5.109, *P* = 0.0238), with a survival pattern distinct from that observed under sugar feeding. A cage-level sensitivity analysis using replicate cages as the experimental unit yielded results consistent with the primary Kaplan-Meier analysis [Supplementary Table 2].

**Figure 1:**
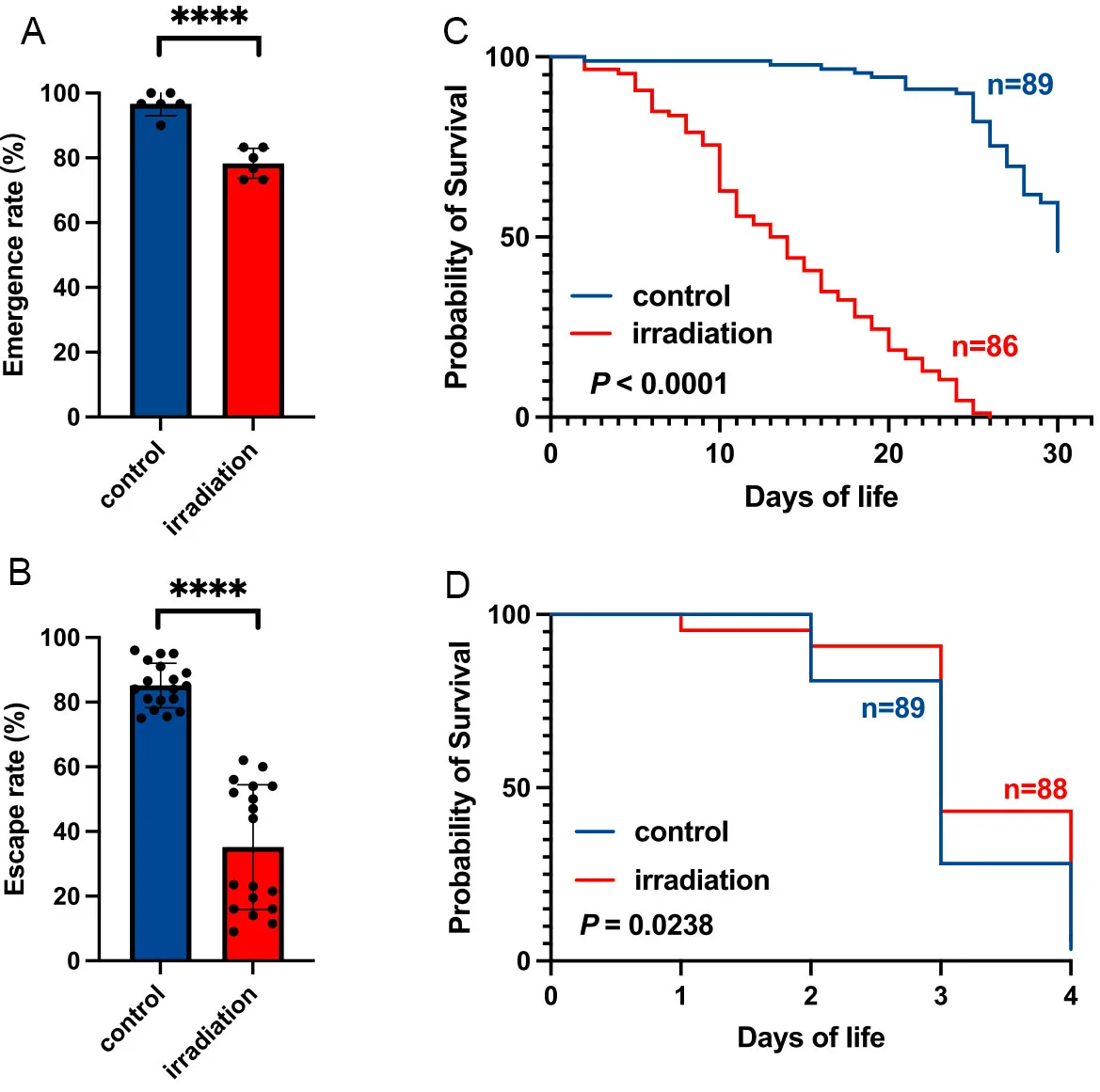
Effects of irradiation on the quality of *Aedes albopictus* GT male mosquitoes. (A) Emergence rate; (B) Flight ability; Data in (A) and (B) are presented as mean ± SD. Each data point represents one biological replicate. Emergence rate and flight ability were analyzed using generalized linear mixed models (GLMMs) with a binomial error distribution. **** indicates that *P* < 0.0001. Male mosquito lifespan when fed with sugar (C) or sterilized water (D). Kaplan-Meier analysis was used to compare the male longevity between irradiated and control males. *P* < 0.05 was considered statistically significant. SD: Standard deviation.

### Irradiation does not alter gut microbiota alpha diversity in males

Comparison of alpha diversity metrics (Chao and Shannon indices) revealed no significant differences between irradiated and control groups at any developmental stage [Figure 2A-E]. Though diversity tended to decrease with age in both groups, PERMANOVA confirmed no statistically significant effects of treatment or age (all *P* > 0.05, Table 1).

**Figure 2:**
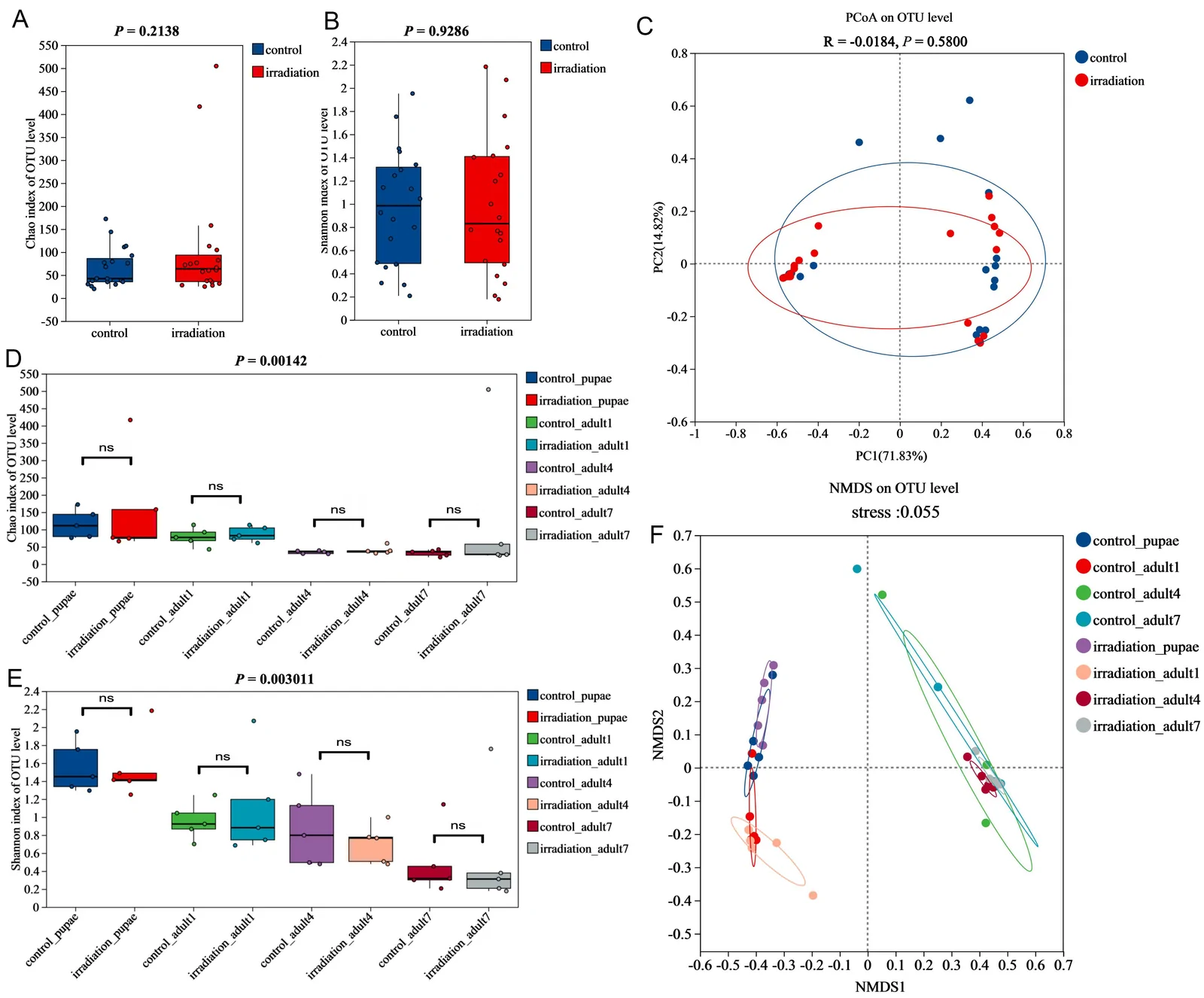
Effects of irradiation on the α- and β-diversity of the gut microbiota in *Aedes albopictus* GT male mosquitoes. (A) Chao diversity index; (B) Shannon diversity index. Student’s *t*-test was used to compare the Chao and Shannon diversity indices. (C) PCoA (principal coordinates analysis) based on weighted UniFrac distance algorithm; (D) Chao diversity index; (E) Shannon diversity index. One-way ANOVA for multiple group comparisons and Student’s t-test for pairwise comparisons; (F) NMDS (non-metric multidimensional scaling) analysis based on Bray-Curtis distance algorithm. *P* < 0.05 represents a statistically significant difference. ns indicates no statistically significant difference. OTU: Operational taxonomic unit; GT: ANOVA: one-way analysis of variance.

**Table 1:** PERMANOVA results for irradiation treatment and irradiation-age combined factors at the genus level

**Characteristics**	**Sums of Sqs**	**Mean Sqs**	**F_Model**	**R2**	***P*_value**	***P*_adjust**
control & irradiation	0.0845	0.0845	0.2934	0.0077	0.8330	0.8330
control_pupae & irradiation_pupae	0.0870	0.0870	1.5440	0.1618	0.1750	0.1750
control_adult1 & irradiation_adult1	0.0675	0.0675	1.372	0.1464	0.2520	0.2520
control_adult4 & irradiation_adult4	0.0656	0.0656	0.7347	0.0841	0.7770	0.7770
control_adult7 & irradiation_adult7	0.1964	0.1964	1.6407	0.1702	0.3630	0.3630

Forty samples were studied, with five biological replicates for each group. The table shows no statistically significant differences among the irradiation treatment and irradiation-age combined factors (all *P* > 0.05). PERMANOVA: Permutational multivariate analysis of variance.

NMDS analysis based on Bray-Curtis dissimilarity suggested a separation trend between irradiated and control samples at the pupal and 1-day-old stages, whereas samples from 4-day-old and 7-day-old males clustered more closely [Figure 2F]. These clustering patterns suggest that irradiation induces stage-specific shifts in gut microbiota composition, with pronounced differences at earlier developmental stages. As mosquitoes age, their gut microbiota appears to stabilize, resulting in reduced variation between the 4-day-old and 7-day-old groups.

### Irradiation affects the relative abundance of major gut microbiota in males

The Circos diagram [Figure 3A], illustrating species relationships at the phylum level, shows that the gut microbiota of GT males is primarily composed of Bacteroidota, Pseudomonadota, and Bacillota. Comparative analysis showed that both irradiated and control males harbored overlapping as well as unique phyla. Specifically, 22 phyla were consistently present in both groups [Figure 3B]. Two phyla, GAL15 (58.3%) and Fibrobacterota (41.7%), were unique to the control group [Figure 3C], while seven phyla, including Elusimicrobiota (30.7%), Dependentiae (21.1%), Armatimonadota (14.9%), Caldatribacteriota (11.4%), NB1-j (7.9%), Deferribacterota (7.0%), and Latescibacterota (7.0%), were unique to the irradiated group [Figure 3D].

**Figure 3:**
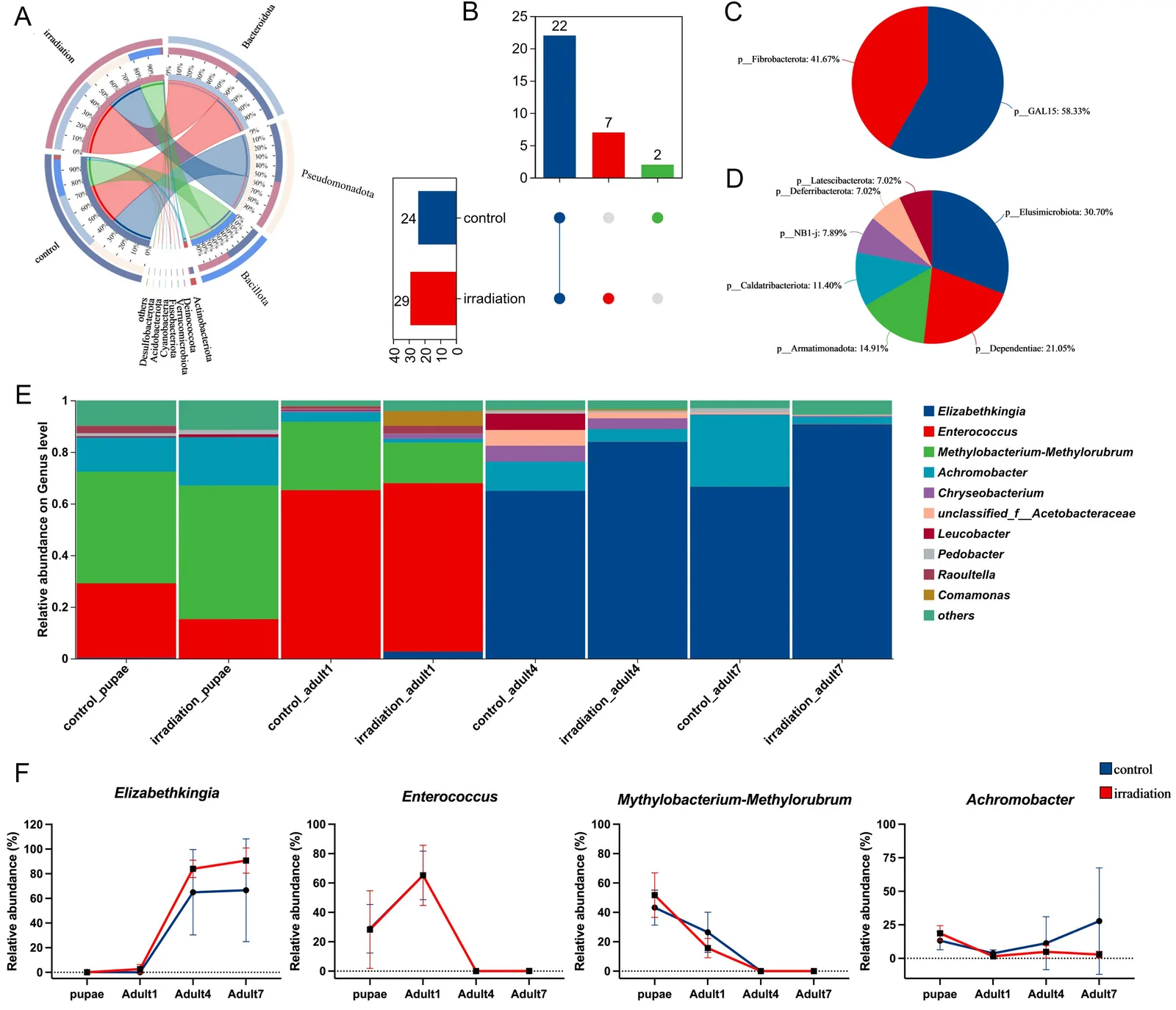
Effects of irradiation on the composition of the gut microbiota in *Aedes albopictus* male mosquitoes. (A) Species relationships Circos diagram (phylum level); (B) Venn diagram of species composition (phylum level); (C) Phylum composition unique to the control group; (D) Phylum composition unique to the irradiated group; (E) Gut microbiota community bar chart at the genus level; (F) Dynamic changes in the relative abundance of high-abundance genera in the gut of GT males at different developmental stages.

Across 40 gut microbiota samples, the relative abundance profile at the genus level revealed that the microbiota composition was strongly affected by mosquito age. The gut microbiota of pupae and 1-day-old mosquitoes were generally similar, dominated by *Enterococcus*, *Methylobacterium-Methylorubrum*, and *Achromobacter*. In contrast, 4-day-old and 7-day-old males shared a different profile, with a predominance of *Elizabethkingia*, *Achromobacter*, and *Chryseobacterium*.

Irradiation primarily affected the relative abundance of bacterial genera. In irradiated pupae, *Enterococcus* abundance decreased, while *Methylobacterium-Methylorubrum* and *Achromobacter* increased. In 1-day-old irradiated males, Comamonas increased, while *Methylobacterium-Methylorubrum* and *Achromobacter* decreased. Among 4-day-old irradiated males, *Elizabethkingia* increased, whereas *Achromobacter* and *Leucobacter* decreased. In 7-day-old irradiated males, *Elizabethkingia* again increased, accompanied by a decline in *Achromobacter* and *Sphingomonas* [Figure 3E].

Dynamic analysis of the four most abundant genera revealed the following distinct trends. *Elizabethkingia* showed the highest relative abundance in 4-day-old and 7-day-old males [Figure 3F], while *Enterococcus* reached its peak at the 1-day-old stage before declining. *Methylobacterium-Methylorubrum* declined significantly with age, eventually disappearing. The abundance of *Achromobacter* varied notably between irradiated and control groups, depending on developmental stage.

### Irradiation reshapes the composition of cultivable microbiota in males

A total of 487 cultivable microbial isolates were analyzed, 204 from the control group and 283 from the irradiated group, to investigate compositional differences in the cultivable microbiota of GT male mosquitoes. At the family level, the gut microbiota of both groups was predominantly composed of *Enterococcaceae* (control: 55.3%; irradiated: 21.1%), *Yersiniaceae* (control: 15.2%; irradiated: 9.5%), *Weeksellaceae* (control: 7.4%; irradiated: 18.5%), and *Comamonadaceae* (control: 5.9%; irradiated: 5.8%) [Figure 4A and B]. Notably, *Microbacteriaceae*, *Aeromonadaceae*, and *Flavobacteriaceae* were only detected in the irradiated group [Supplementary Table 3]. Additionally, three fungal families, *Aspergillaceae*, *Debaryomycetaceae*, and *Rhynchogastremaceae*, were also cultured from the gut microbiota of both groups, further highlighting the microbial diversity in the mosquito gut [Supplementary Table 3].

**Figure 4:**
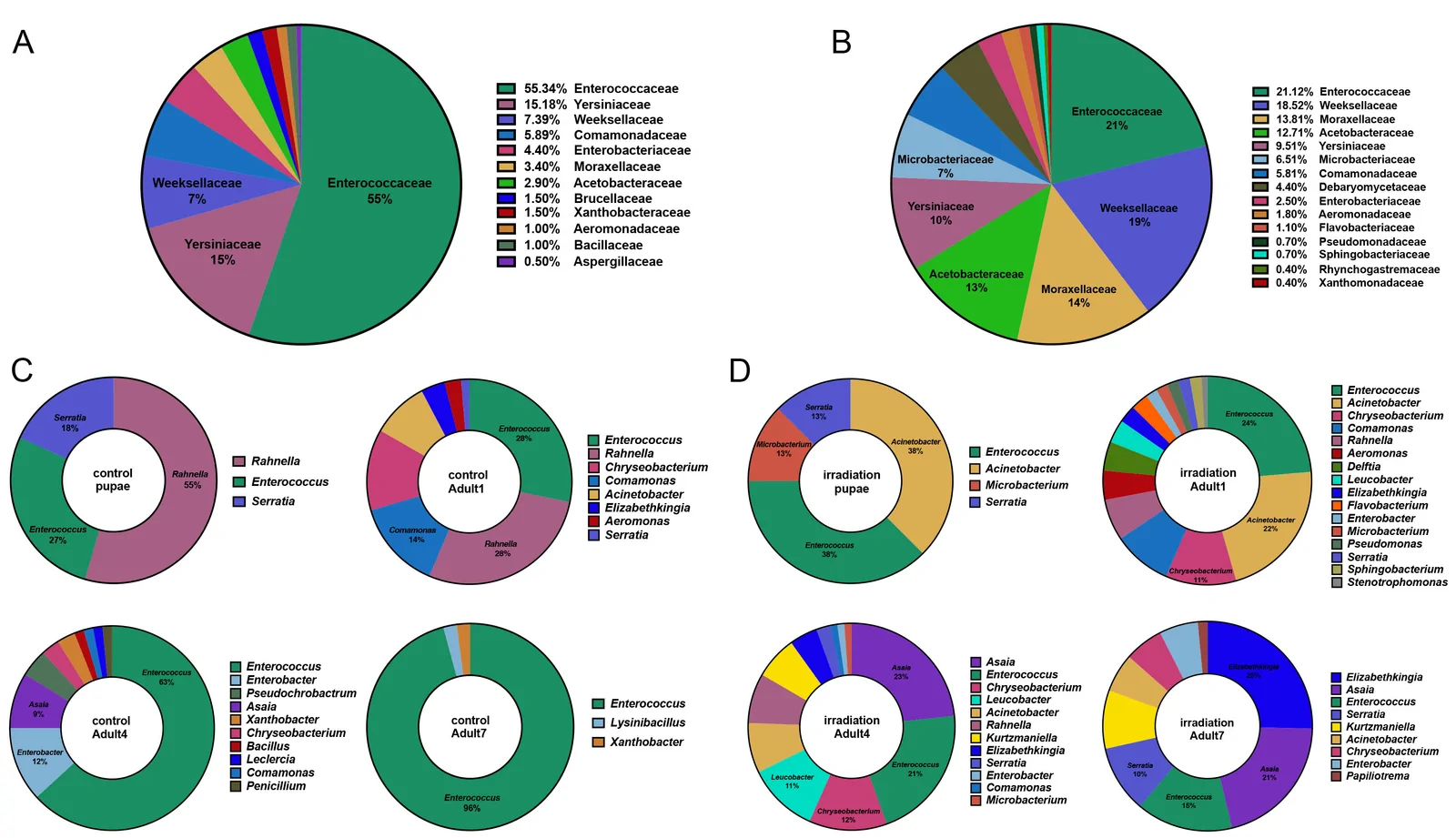
Composition of the cultivable gut-associated microbiota of *Aedes albopictus* GT male mosquitoes with and without irradiation; (A) Cultivable gut microbiota composition (family level) without irradiation; (B) Cultivable gut microbiota composition (family level) after irradiation; (C) Cultivable gut microbiota composition in the control males at different ages (genus level); (D) Cultivable gut microbiota composition in the irradiated males at different ages (genus level).

Genus-level analysis across developmental stages revealed distinct age- and treatment-related patterns. In the control male group, dominant genera included *Rahnella* (54.5%), *Enterococcus* (27.3%), and *Serratia* (18.2%) [Figure 4C]. In contrast, irradiated pupae harbored *Acinetobacter* (37.5%), *Enterococcus* (37.5%), *Serratia* (12.5%), and *Microbacterium* (12.5%) [Figure 4D].

At 1 day old, the control group was dominated by *Rahnella* (28.2%), *Enterococcus* (28.2%), *Comamonas* (14.1%), and *Chryseobacterium* (12.8%). In irradiated 1-day-old groups, *Enterococcus* (23.7%), *Acinetobacter* (21.9%), *Flavobacterium* (2.7%), and *Comamonas* (9.1%) were dominant.

At 4 days old, the microbiota of the control samples was mainly composed of *Enterococcus* (63.2%), *Enterobacter* (11.8%), and *Asaia* (8.8%). In the irradiated group, the community was more diverse, including *Asaia* (23.3%), *Enterococcus* (21.1%), *Chryseobacterium* (12.2%), *Leucobacter* (11.1%), *Acinetobacter* (7.8%), and *Rahnella* (7.8%).

At 7 days old, *Enterococcus* dominated the control group (95.8%), while the irradiation group showed a more heterogeneous profile, comprising *Elizabethkingia* (25.4%), *Asaia* (20.9%), *Enterococcus* (14.9%), and *Serratia* (10.4%).

In the control group, *Enterococcus* increased with age, while *Rahnella* and *Serratia* decreased. In contrast, irradiation resulted in greater bacterial diversity across stages, though *Enterococcus* remained a consistently abundant genus.

### Significant differences in gut-associated bacterial communities between irradiated and control groups

To identify further significant differences in gut-associated bacterial communities between groups, we performed an in-depth analysis using the Linear Discriminant Analysis Effect Size (LEfSe) method. An LDA score threshold of > 2 was used to determine significantly enriched taxa [Figure 5A]. These results were combined with inter-group comparison tests [Figure 5B], revealing distinct microbial signatures in the irradiated group. Notably, the genera *Delftia* and *Stenotrophomonas* exhibited significant shifts in abundance. Further age-specific analysis showed that *Delftia* was significantly more abundant in irradiated pupae compared to controls [Figure 5C]. In 1-day-old males, *Achromobacter* significantly decreased post-irradiation, whereas *Stenotrophomonas* increased [Figure 5D]. No significant genus-level differences were observed in 4-day-old irradiated and control groups. The abundance of *Comamonas* and *Sphingobacterium* was significantly higher in the irradiated group compared to the control group [Figure 5E]. To further explore temporal patterns, we examined the relative abundance of selected culturable genera that were previously identified as differentially abundant between groups. The abundance of these genera was visualized across developmental stages [Figure 5F]. While no statistical comparisons were performed for these temporal profiles, several genera, including *Stenotrophomonas*, *Comamonas*, and *Sphingobacterium*, showed apparent increases following irradiation and subsequent declines over time. Similarly, *Delftia* and *Achromobacter* displayed higher relative abundance at early stages, followed by gradual decreases as mosquitoes aged. These observations provide a descriptive overview of potential temporal dynamics of irradiation-associated bacterial taxa.

**Figure 5:**
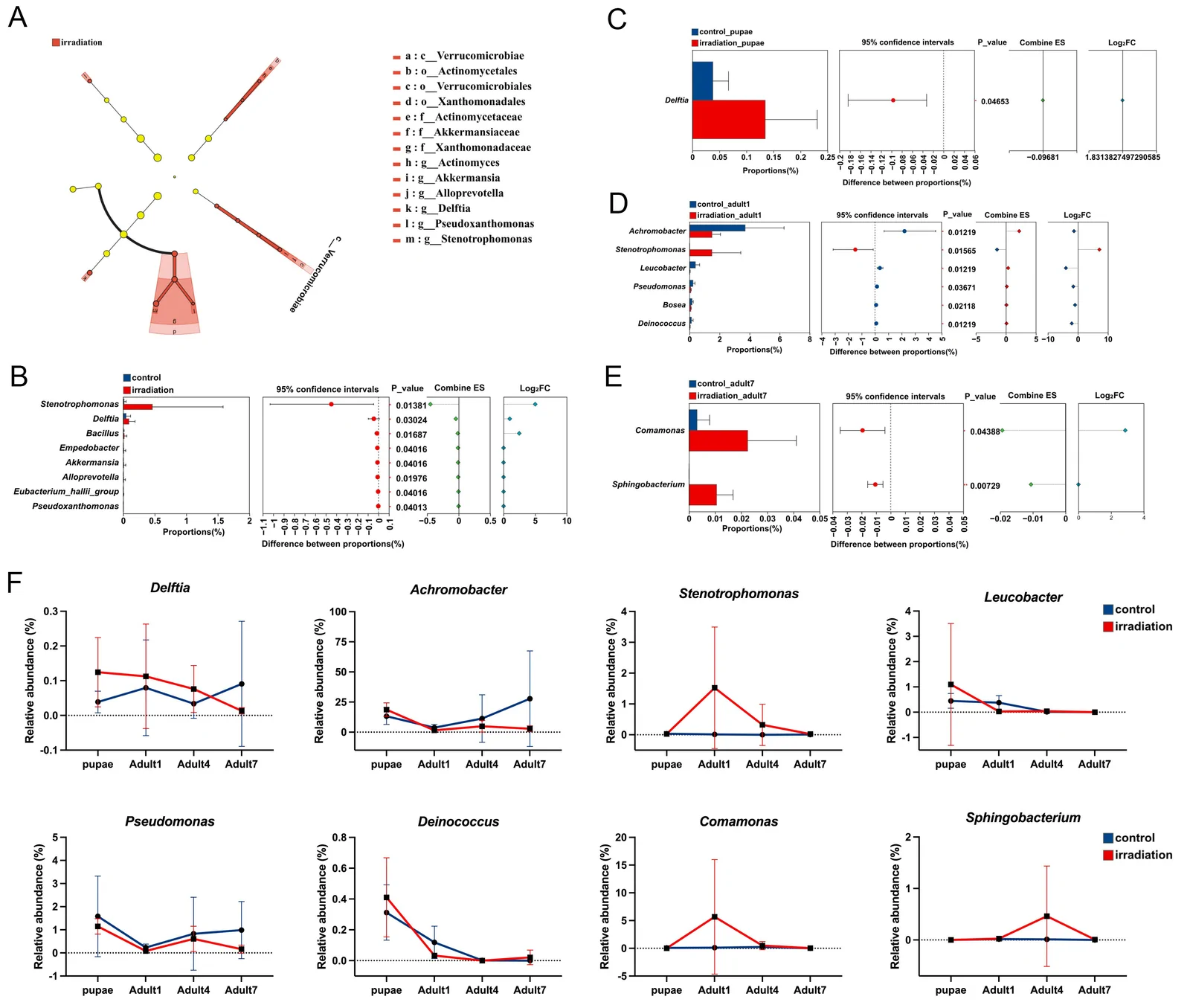
LDA of the cultivable gut-associated bacterial species of *Aedes albopictus* GT male mosquitoes with and without irradiation. (A) Indicator bacteria with LDA scores of 2 or greater in bacterial communities associated with male mosquito guts in irradiated and control groups; (B) Comparison of genus-level relative abundance between irradiated and control groups; (C) Comparison of genus-level relative abundance between irradiated and control pupae; (D) Comparison of genus-level relative abundance between irradiated and control one-day-old adults; (E) Comparison of genus-level relative abundance between irradiated and control seven-day-old adults; (F) Line chart showing the dynamic changes in the relative abundance of bacterial genera that were significantly different between irradiated and control samples and successfully isolated through culture-based methods at different ages. Statistical significance in panels (B-E) was assessed using the two-tailed Mann-Whitney test. Data in panel (F) are presented as mean ± SD. SD: Standard deviation; LDA: linear discriminant analysis.

To further validate the irradiation-associated shifts detected in the 16S *rRNA* sequencing analysis, the relative abundance of the eight culturable genera was quantified by qPCR [Figure 6]. In pupae, *Delftia* [Figure 6A] exhibited a higher relative abundance in the irradiated group than in the control group, consistent with its sequencing-derived enrichment at this stage. In one-day-old males, the relative abundance of *Achromobacter* [Figure 6B], *Leucobacter* [Figure 6C], *Pseudomonas* [Figure 6D], and *Deinococcus* [Figure 6E] was markedly reduced in irradiated individuals, supporting the sequencing-based depletion of these taxa shortly after adult emergence. Conversely, *Stenotrophomonas* [Figure 6F] showed an increased relative abundance in irradiated one-day-old males, consistent with its irradiation-associated elevation observed in the sequencing analysis. In contrast, in seven-day-old males, *Comamonas* [Figure 6G] and *Sphingobacterium* [Figure 6H] were significantly more abundant in irradiated mosquitoes, matching the stage-specific increases observed in the sequencing dataset. Overall, the qPCR results confirmed the irradiation-responsive patterns identified by the 16S *rRNA* gene analysis and validated these eight taxa as key differential genera affected by pupal irradiation.

**Figure 6:**
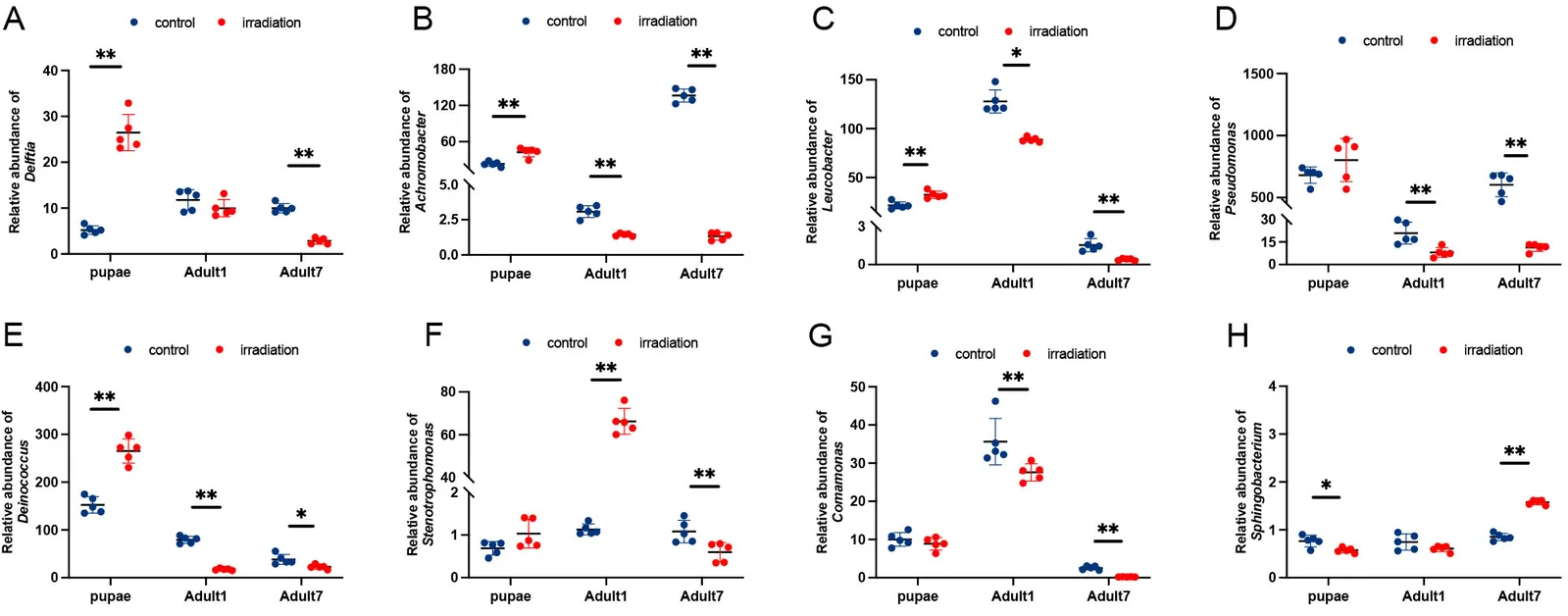
qPCR validation of eight differential gut bacterial genera in irradiated and control *Aedes albopictus* GT males. Panels (A-H) show the relative abundance of *Delftia* (A), *Achromobacter* (B), *Leucobacter* (C), *Pseudomonas* (D), *Deinococcus* (E), *Stenotrophomonas* (F), *Comamonas* (G), and *Sphingobacterium* (H) in the guts of irradiated and control males at different developmental stages (pupae, 1-day-old adults, and 7-day-old adults). Data are represented as mean ± SD. Statistical significance between irradiated and control groups is indicated (* indicates that *P* < 0.05, ** indicates that *P* < 0.01). *P* < 0.05 represents a statistically significant difference. SD: Standard deviation.

### Functional prediction and physiological validation of changes in gut-associated bacterial communities following irradiation

Functional prediction based on 16S *rRNA* gene sequencing revealed a decreased abundance of enzymes 6.3.5.7 (glutaminyl-tRNA synthetase, glutamine hydrolyzing) and 6.3.5.6 (asparaginyl-tRNA synthetase, glutamate hydrolyzing) in irradiated male mosquito groups [Figure 7A]. In response, glutamine synthetase (GS) activity was measured across four age groups. Compared to the control, GS activity was significantly reduced in irradiated groups at the pupal stage (57.37 ± 3.44 *vs.* 38.34 ± 5.19, μmol/h/g, *P* = 0.0022), four-day-old group (36.36 ± 3.41 *vs.* 29.72 ± 3.17, μmol/h/g, *P* = 0.0108), and the seven-day-old group (13.50 ± 3.84 *vs.* 8.61 ± 2.57, μmol/h/g, *P* = 0.0260), but not at one-day-old group (44.64 ± 2.73 *vs.* 45.12 ± 4.32, μmol/h/g, *P* = 0.8733) [Figure 7B].

**Figure 7:**
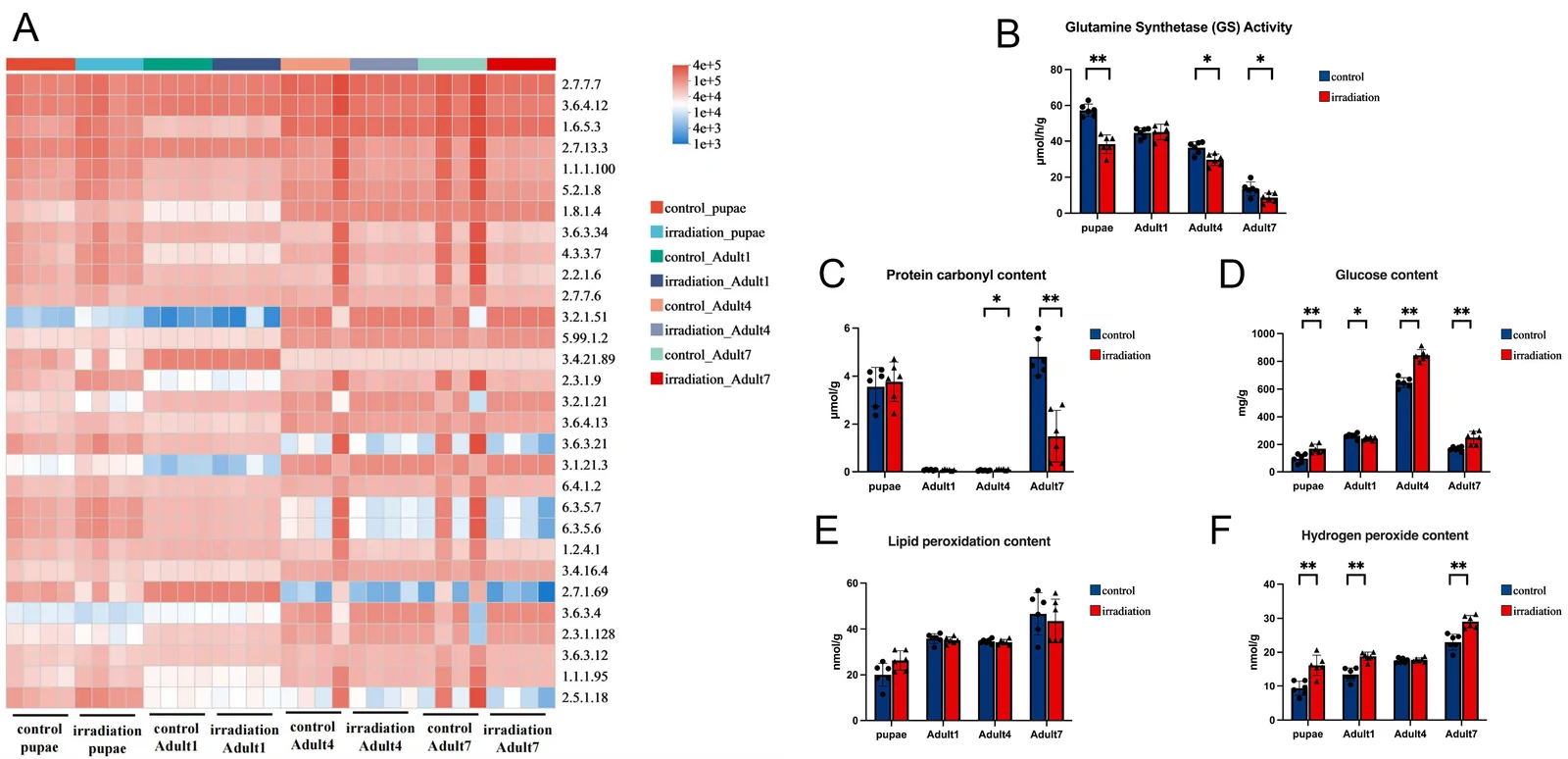
Functional prediction and metabolic differences in irradiated and non-irradiated male mosquitoes. (A) Functional prediction of gut microbiota using PICRUSt2; (B) Glutamine synthetase activity; (C) Protein carbonyl content; (D) Glucose content. (E) Lipid peroxidation level; (F) Hydrogen peroxide content. All metabolic parameters were measured using commercial assay kits. Data are presented as mean ± SD. Mann-Whitney test was used to compare the metabolic parameters between control and irradiated groups. * indicates *P* < 0.05, ** indicates *P* < 0.01, represents statistically significant difference. SD: Standard deviation.

Protein carbonyl content showed age-dependent responses to irradiation across developmental stages. No significant difference was observed between irradiated and control groups at the pupal stage (3.56 ± 0.81 *vs.* 3.77 ± 0.83, *P* = 0.6147) or in the one-day-old group (0.076 ± 0.020 *vs.* 0.079 ± 0.026, μmol/g, *P* = 0.9242). However, content significantly increased in the irradiated four-day-old group (0.055 ± 0.019 *vs.* 0.098 ± 0.024, μmol/g, *P* = 0.0130) and significantly decreased in the seven-day-old group (4.81 ± 0.78 *vs.* 1.50 ± 1.08, μmol/g, *P* = 0.0022) [Figure 7C].

Glucose content was significantly elevated in irradiated males across most age groups, indicating disrupted glucose metabolism. Compared to controls, irradiated mosquitoes had higher glucose levels at the pupal stage (97.64 ± 33.41 *vs.* 169.71 ± 32.07, mg/g, *P* = 0.0022), four-day-old (647.00 ± 35.65 *vs.* 844.23 ± 41.04, mg/g, *P* = 0.0022), and seven-day-old stages (169.09 ± 16.35 *vs.* 250.09 ± 45.51, mg/g, *P* = 0.0022). Interestingly, glucose content was significantly lower in irradiated one-day-old males (263.37 ± 19.26 *vs.* 243.01 ± 12.10, mg/g, *P* = 0.0368) [Figure 7D].

Lipid peroxidation levels did not differ significantly between irradiated and control groups at any developmental stage [Figure 7E].

Hydrogen peroxide (H_2_O_2_) levels were significantly elevated in irradiated males at most developmental stages. Compared to controls, irradiated individuals exhibited higher H_2_O_2_ levels at the pupal stage (9.36 ± 2.12 *vs.* 16.16 ± 2.99, nmol/g, *P* = 0.0087), one-day-old stage (13.49 ± 1.89 *vs.* 18.82 ± 1.23, nmol/g, *P* = 0.0022), and seven-day-old group (22.99 ± 2.31 *vs.* 29.04 ± 1.81, nmol/g, *P* = 0.0022). However, no significant difference was observed in the four-day-old group (17.57 ± 0.66 *vs.* 17.79 ± 0.60, nmol/g, *P* = 0.5108) [Figure 7F].

## DISCUSSION

Mosquitoes are major human pathogen vectors^[28-30]^, and integrated control measures are essential, with the SIT playing a promising role^[5]^. Irradiation, a common sterilization method for male mosquitoes, acts as a controlled physiological stressor to evaluate irradiation-induced quality loss.

In this study, we irradiated mass-reared male *Ae. albopictus* to induce sterility and systematically assess its effects on mosquito quality and gut microbiota. Our core objective was to examine whether gut microbiota profiles respond consistently to irradiation and are associated with irradiation-associated quality loss. We observed significant lifespan reduction, and reproducible gut microbiota shifts after irradiation, which supports the potential of gut microbiota signatures as informative features associated with sterile males’ physiological status and quality. Dominant phyla in mass-reared mosquitoes included Bacteroidota, Pseudomonadota, and Bacillota, with core genera such as *Achromobacter* and *Pseudomonas* stably present across life stages. In contrast, wild *Ae. albopictus*, particularly larval stages, harbored more diverse microbiota^[13,15]^, including Verrucomicrobiota, Spirochaetota, and bacterial families such as *Sphingomonadaceae*, *Spirosomaceae*, and *Chitinophagaceae*. Unique genera like *Dietzia*, *Neisseria*, and *Hydrogenophaga* were found exclusively in wild populations^[15]^. Both groups shared functionally relevant taxa (*Enterococcus*, *Elizabethkingia*) with different abundance patterns. These contrasts confirm that rearing conditions shape mosquito microbiota, underscoring the need to account for microbial background when comparing laboratory and field physiological/ecological outcomes^[13,15,31]^. Additionally, 16S *rRNA* sequencing confirmed the absence of *Wolbachia* in our mass-reared strain [Figure 3E], which is used as a robust biomarker to distinguish released sterile males from wild populations, facilitating field monitoring of SIT effectiveness^[19]^.

Gut bacterial community structure varied with mosquito age, reflecting dynamic developmental restructuring. Pupae and 1-day-old males exhibited similar bacterial profiles dominated by *Enterococcus*, *Methylobacterium-Methylorubrum*, and *Achromobacter*, while 4- and 7-day-old males showed shifts toward *Elizabethkingia*, *Chryseobacterium*, and other Bacteroidota-associated taxa. These age-associated changes suggest progressive microbiota maturation, likely driven by dietary, physiological, or immune shifts during development^[32,33]^. Notably, *Achromobacter* and *Pseudomonas* were consistently detected at all stages, indicating their potential role as stable community members. Such stage-specific microbial patterns may provide reference profiles for assessing the developmental status and physiological consistency of mass-reared males^[34,35]^. Furthermore, taxa identified here may represent candidate targets for future microbiota-based interventions aimed at improving sterile male performance.

Irradiation significantly altered gut microbiota composition, which was associated with physiological impairment. Several anaerobic bacterial phyla, including Elusimicrobiota, Dependentiae, Deferribacterota, and Latescibacterota, emerged after irradiation and were absent in controls, which may reflect irradiation-induced tissue damage or gut hypoxia that favors anaerobes^[8,36]^. Notably, irradiation significantly reduced potentially beneficial taxa involved in nutrient assimilation and detoxification^[37]^, potentially impairing host metabolism. These shifts suggest that irradiation may affect gut-associated bacterial communities through multiple mechanisms, including indirect host-mediated effects such as epithelial barrier disruption, immune modulation, and altered nutrient availability, as well as potential direct effects on bacteria, for example through radiation-induced DNA damage or selective pressure on microbial populations^[38-40]^. qPCR confirmed the irradiation-associated abundance patterns of these taxa, validating stage-specific shifts in the eight culturable genera identified by 16S *rRNA* analysis. This independent verification strengthens our microbiota findings and confirms the consistency of irradiation-associated gut bacterial alterations. To explore microbial impacts on host metabolism, we measured key physiological indicators: irradiation reduced glutamine synthetase activity and increased glucose/hydrogen peroxide levels, indicating disrupted amino acid metabolism, impaired glucose utilization, and elevated oxidative stress. Glutamine plays a central role in insect nitrogen homeostasis^[41]^. Their ammonia detoxification system depends on glutamine synthetase and glutamate dehydrogenase^[42-44]^. These findings suggest that irradiation-associated microbiota dysbiosis coincides with metabolic changes that may be related to sterile male mosquito quality.

An additional explanation for the stage-specific patterns observed in this study is radiation-induced precocity. Previous studies have shown that irradiation can accelerate physiological and behavioral maturation in mosquitoes and other insects, allowing irradiated males to exhibit earlier onset of adult traits. For example, Bellini *et al.* reported that irradiated *Aedes albopictus* males displayed earlier mating activity, conferring a temporary competitive advantage^[45]^. Such shifts in developmental timing may help explain the heterogeneous responses observed across age groups in the present study. Rather than reflecting uniform reductions in physiological performance, some of the observed changes - such as the absence of GS differences at the one-day stage and the dynamic restructuring of gut-associated bacterial communities - may instead indicate altered temporal trajectories of development. This perspective suggests that irradiation may not only impair biological functions but also shift their timing, leading to apparent inconsistencies across developmental stages. Radiation-induced precocity may therefore provide a more integrated framework for interpreting both microbiota and physiological changes observed in irradiated males.

Besides the expected reductions in irradiated males’ emergence, flight ability and sugar-fed longevity, we observed an opposite phenomenon: all males died within 96 h, but irradiated males had a transient 48-72 h survival advantage when fed sterile distilled water without sugar. This short-lived effect may reflect hormesis-like preconditioning^[46]^. Thus, male quality is nutrition-dependent: irradiated males perform poorly under normal feeding conditions but exhibit brief resilience during starvation, which does not persist^[47]^. Operationally, SIT programs must provide sugar before release; starvation’s temporary benefits do not offset deficits in other quality parameters (e.g., mating-related traits)^[5]^.

Beyond identifying gut microbiota profiles associated with irradiation-induced quality impairment in mass-reared *Ae. albopictus* males, our findings also open new perspectives for improving sterile male performance prior to release. Several of the bacterial taxa affected by irradiation in this study are culturable and therefore suitable for targeted manipulation during mass-rearing or post-irradiation diet supplementation. Previous SIT studies showed beneficial gut bacteria can partially restore irradiation-induced quality loss: probiotics recovered ecological fitness in *Bactrocera dorsalis*^[17]^ and microbiota manipulation improved mating competitiveness in mass-reared Ceratitis capitata males^[48]^. Combined with these studies, our results suggest gut microbiota profiling may have potential as a quality assessment approach and provide a foundation for developing probiotic-based approaches to restore or enhance sterile male biological quality.

A limitation of this study is the discrepancy between the nominal irradiation dose and the actual absorbed dose resulting from the non-filtered X-ray configuration. Although the irradiation protocol was originally designed to deliver a nominal dose of 30 Gy, subsequent dosimetric verification using radiochromic film demonstrated that the actual absorbed dose delivered to the mosquito pupae was 78 Gy (95% CI: 74.1-81.9 Gy). Therefore, all biological results presented here should be interpreted as responses to this verified absorbed dose rather than the intended nominal dose. Nevertheless, previous experiments using the same mosquito strain demonstrated more than 99% sterility at an absorbed dose of approximately 30 Gy^[19]^, indicating that the primary objective of the present study was not to establish a dose-response relationship, but rather to characterize microbiota and physiological changes under the irradiation conditions that were actually delivered. In addition, although irradiation-associated microbiota shifts were consistently associated with physiological changes, the present study does not establish a direct causal relationship between microbiota alterations and sterile male quality loss. Future studies involving microbiota manipulation and functional validation will be required to determine whether specific bacterial taxa contribute directly to irradiation-associated changes in mosquito quality^[17]^.

Overall, this study demonstrates that irradiation-induced sterile male quality loss is accompanied by reproducible, quantifiable shifts in gut microbiota composition and metabolic status. Rather than viewing these microbial changes solely as detrimental effects, we highlight gut microbiota profiles as informative microbial signatures associated with irradiation-induced physiological impairment. Incorporating microbiota-based indicators into SIT quality control frameworks can enhance the assessment of sterile male quality and support the optimization of mosquito control programs.

## DECLARATIONS

### Availability of data and materials

The datasets used and analyzed during the current study are available from the corresponding author upon reasonable request.

### Copyright

© The Author(s) 2026.
