# Supplementary material for: Gut microbiota profiles as potential indicators of irradiation-induced quality loss in mass-reared Aedes albopictus males
Source: Microbiome Res Rep. 2026 Aug 28;5(3):19. doi: 10.20517/mrr.2026.25 (PMC13678544; doi:10.20517/mrr.2026.25)
Supplement: Supplementary file 1 [file mrr-5-3-19-SupplementaryMaterials.pdf]

Microbiome Research Reports

Supplementary Materials

Gut microbiota profiles as potential indicators of irradiation-induced quality loss in mass-reared *Aedes albopictus* males

Paerhande Dilinuer[1,2,3], Jehangir Khan[1,2,3,4], Ming Li[1,2,3], Yu Wu[1,2,3], Xiaoying Zheng[1,2,3], Zhongdao Wu[1,2,3], Datao Lin[1,2,3], Hanano Yamada[5], Jeremy Bouyer[6], Kostas Bourtzis[5], Dongjing Zhang[1,2,3]

[1]Chinese Atomic Energy Agency Center of Excellence on Nuclear Technology Applications for Insect Control, Key Laboratory of Tropical Disease Control of the Ministry of Education, Sun Yat-sen University, Guangzhou 510080, Guangdong, China.

[2]International Atomic Energy Agency Collaborating Centre, Sun Yat-sen University, Guangzhou 510080, Guangdong, China.

[3]Guangdong Provincial Engineering Technology Research Center for Diseases-Vectors Control, Sun Yat-sen University, Guangzhou 510080, Guangdong, China.

[4]Zoology Department, Abdul Wali Khan University Mardan, Khyber Pakhtunkhwa 23200, Pakistan.

[5]Insect Pest Control Section, Joint FAO/IAEA Centre of Nuclear Techniques in Food and Agriculture, Department of Nuclear Sciences and Applications, International Atomic Energy Agency, Vienna 1400, Austria.

[6]ASTRE, Cirad, INRAE, Université de Montpellier, Plateforme Technologique CYROI, Sainte-Clotilde, La Réunion 97490, France.

© The Author(s) 2021. Open Access This article is licensed under a Creative Commons Attribution 4.0 International License (https://creativecommons.org/licenses/by/4.0/), which permits unrestricted use, sharing, adaptation, distribution and reproduction in any medium or

format, for any purpose, even commercially, as long as you give appropriate credit to the original author(s) and the source, provide a link to the Creative Commons license, and indicate if changes were made.

OAE Publishing Inc.

https://www.oaepublish.com/mrr

Correspondence to: Dr. Dongjing Zhang, Chinese Atomic Energy Agency Center of Excellence on Nuclear Technology Applications for Insect Control, Key Laboratory of Tropical Disease Control of the Ministry of Education, Sun Yat-sen University, Guangzhou 510080, Guangdong, China; Chinese Atomic Energy Agency Center of Excellence on Nuclear Technology Applications for Insect Control, Key Laboratory of Tropical Disease Control of the Ministry of Education, Sun Yat-sen University, Guangzhou 510080, Guangdong, China; Guangdong Provincial Engineering Technology Research Center for Diseases-Vectors Control, Sun Yat-sen University, Guangzhou 510080, Guangdong, China. E-mail: zhangdj5@mail.sysu.edu.cn

ORCID: Dongjing Zhang (0000-0002-3960-1036)

Supplementary Table 1. Primer sequences and annealing temperatures used for bacterial qPCR assays

Bacterial taxon	Primer name	Sequence (5'-3')	Annealing temperature (°C)
Delftia	Delftia-F	5'-AAAGCTTACCAAGCCGACGA-3'	55.3
	Delftia-R	5'-CAAAATTCCCCACTGCTGCC-3'	57.35
Achromobacter	Achrom-F	5'-TTGTAAAGCACTTTTGGCAG-3'	56
	Achrom-R	5'-CCAGTAATTCCGATTAACGC-3'	58
Stenotrophomonas	Ste-F	5'-CCTTCGGGAACTCGAACACA-3'	59.8
	Ste-R	5'-AGGGCCATGATGACTTGACG-3'	59.8
Leucobacter	Leucob-F	5'-GTGAGGTAGTGGCTCACCAA-3'	57.35
	Leucob-R	5'-AGAGGTTTACAACCCGAAGGC-3'	57.59

Pseudomonas	Pseu-F	5'-ATACAGAGGGTGCAAGCGTT-3'	54.93
	Pseu-R	5'-GGATGCAGTTCCCAGGTTGA-3'	53.25
Deinococcus	Deicoc-F	5'-AGCGTTACCCGGAATCACTG-3'	57.35
	Deicoc-R	5'-TCCAGAGGTCAAGACACCCA-3'	57.35
Comamonas	Comam-F	5'-GAGTAGCTAATACCGCATGAGA-3'	55.94
	Comam-R	5'-TCCTCTCAGACCAGCTACAG-3'	57.35
Sphingobacterium	Sph-F	5'-GATGGGCTCGCGTGACATTA-3'	57.35
	Sph-R	5'-AGTCTGGTCCGTGTCTCAGT-3'	57.35

Supplementary Table 2. Cage-level restricted mean survival time (RMST) analysis

**Condition**	**Control cage1**	**Control cage2**	**Control cage3**	**Irradiation cage1**	**Irradiation cage2**	**Irradiation cage3**	***P***
sugar	28.00	28.10	27.00	13.69	13.68	14.41	< 0.0001
sterilized water	3.03	3.00	3.23	3.34	3.21	3.33	0.0840

Restricted mean survival time (RMST) was calculated for each replicate cage. P values were obtained using Welch's *t*-test.

Supplementary Table 3. List of cultivable gut bacterial and fungal taxa of irradiated and control *Aedes albopictus* males, including their taxonomic classification (phylum to genus), culture media, and isolation sources

**Phylum**	**Class**	**Order**	**Family**	**Genus**	**Medium**	**Resource**

Proteobacteria	Alphaproteobacteria	Hyphomicrobiales	*Xanthobacteraceae*	*Xanthobacter*	NA	CON: Adult4/Adult7
			Brucellaceae	*Pseudochrobactrum*	TSA/LB/CBA	CON: Adult4
		Rhodospirillales	*Acetobacteraceae*	*Asaia*	CBA/NA/LB/TSA/BHI	CON: Adult4; Irra: Adult4/Adult7
	Betaproteobacteria	Burkholderiales	*Comamonadaceae*	*Delftia*	NA/LB	Irra: Adult1
				Comamonas	CBA/NA/LB/TSA/BHI	CON: Adult1/Adult4; Irra: Adult1/Adult4
	Gammaproteobacteria	Enterobacterales	*Yersiniaceae*	*Rahnella*	CBA/NA/LB/TSA/BHI	CON: pupae/Adult1/Adult4/Adult7; Irra: pupae/Adult1/Adult4/Adult7
				Serratia	CBA/NA/LB/TSA/BHI	CON: pupae/Adult1; Irra: pupae/Adult1/Adult4/Adult7
			Enterobacteriaceae	*Enterobacter*	CBA/NA/LB/TSA/BHI	CON: Adult4; Irra: Adult1/Adult4/Adult7
				Leclercia	BHI	CON: Adult4
		Aeromonadales	*Aeromonadaceae*	*Aeromonas*	CBA/NA/BHI	CON: Adult1; Irra: Adult1
		Moraxellales	*Moraxellaceae*	*Acinetobacter*	CBA/NA/LB/TSA/BHI	CON: Adult1; Irra: pupae/Adult1/Adult4/Adult7

		Xanthomonadales	*Xanthomonadaceae*	*Stenotrophomonas*	BHI	Irra: Adult1
		Pseudomonadales	*Pseudomonadaceae*	*Pseudomonas*	NA	Irra: Adult1
Firmicutes	Bacilli	Bacillales	*Bacillaceae*	*Bacillus*	BHI	CON: Adult4
				Lysinibacillus	LB	CON: Adult7
		Lactobacillales	*Enterococcaceae*	*Enterococcus*	CBA/NA/LB/TSA/BHI	CON: pupae/Adult1/Adult4/Adult7; Irra: pupae/Adult1/Adult4/Adult7
Bacteroidota	Sphingobacteriia	Sphingobacteriales	*Sphingobacteriaceae*	*Sphingobacterium*	NA	Irra: Adult1
	Flavobacteriia	Flavobacteriales	*Flavobacteriaceae*	*Flavobacterium*	NA/TSA	Irra: Adult1
			Weeksellaceae	*Chryseobacterium*	CBA/NA/LB/TSA/BHI	CON: Adult1/Adult4; Irra: Adult1/Adult4/Adult7
				Elizabethkingia	CBA/NA/LB/TSA/BHI	CON: Adult1; Irra: Adult1/Adult4/Adult7
Actinomycetota	Actinomycetes	Mycobacteriales	*Gordoniaceae*	*Gordonia*	NA	Irra: Adult4
				Microbacterium	NA/LB	Irra: pupae/Adult1/Adult4

		Micrococcales	*Microbacteriaceae*	*Leucobacter*	CBA/NA/LB/BHI	Irra: Adult1/Adult4
Ascomycota	Eurotiomycetes	Eurotiales	*Aspergillaceae*	*Penicillium*	LB	CON: Adult4
	Saccharomycetes	Saccharomycetales	*Debaryomycetaceae*	*Kurtzmaniella*	CBA/NA/LB/BHI	Irra: Adult4/Adult7
Basidiomycota	Tremellomycetes	Tremellales	*Rhynchogastremaceae*	*Papiliotrema*	BHI	Irra: Adult4

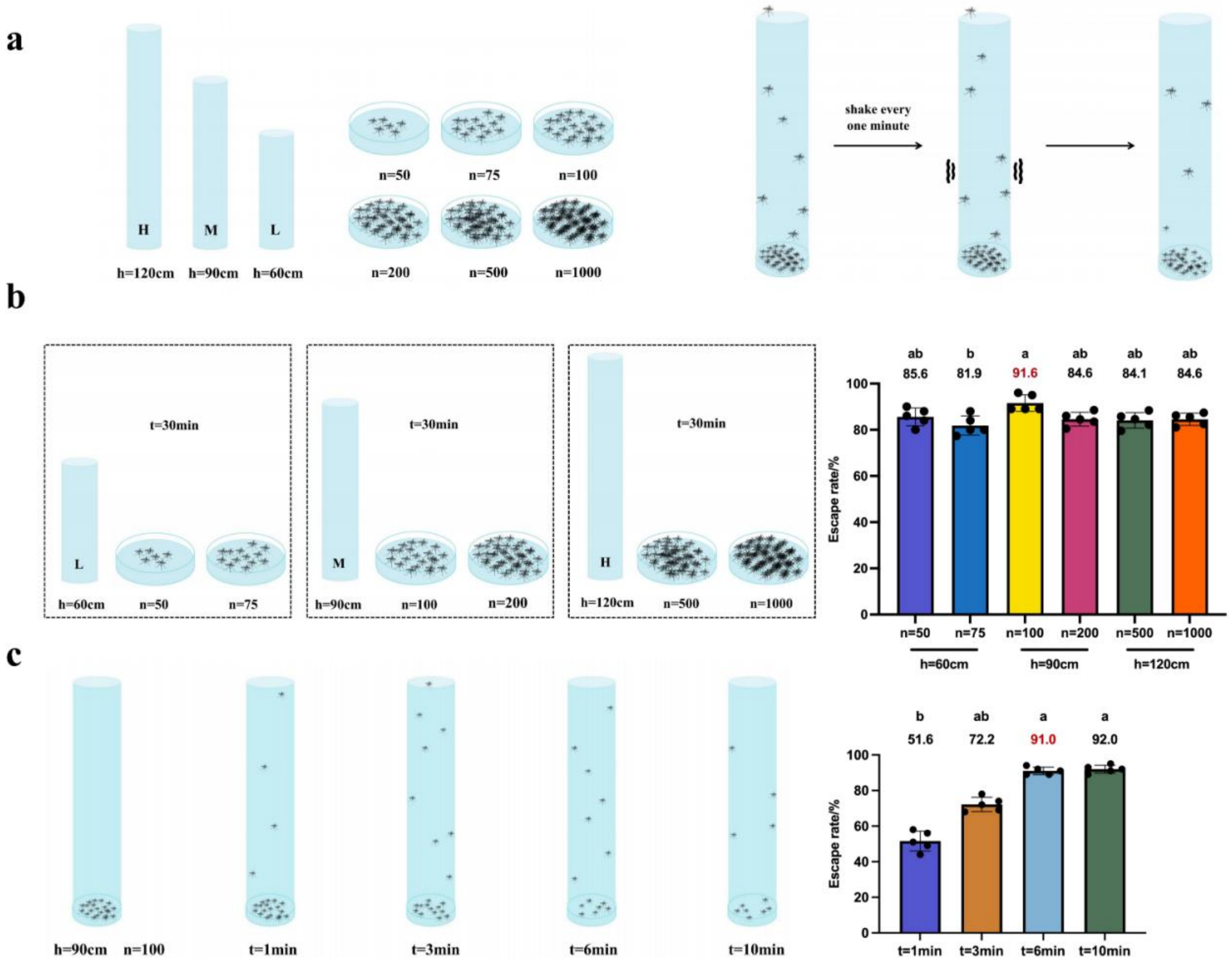

Supplementary Figure 1. Optimization on the flight ability evaluation for male *Aedes albopictus*. (a) Experimental setup. Transparent flight cylinders of different heights (60 cm, 90 cm, and 120 cm) were tested using mass-reared male *Aedes albopictus* at varying mosquito densities per Petri dish (50, 75, 100, 200, 500, and 1000 males). The number of individuals was manually counted in a cooling room with temperature set at 10-12 °C. During the test, the flight cylinder was gently shaken for 30 seconds approximately every 2 min to stimulate movement. (b)

Determination of optimal mosquito density and cylinder height: Mosquitoes from dishes with 50 and 75 males were tested in the 60 cm cylinder, those with 100 and 200 males in the 90 cm cylinder, and those with 500 and 1000 males in the 120 cm cylinder. For each combination, five biological replicates were conducted, and the escape rate was measured after 30 min. Statistical analysis was performed using the Kruskal-Wallis test to compare differences in escape rates across groups. (c) Determination of optimal release duration: Under the condition of 100 males per dish in a 90 cm high cylinder, escape rates were recorded at 1, 3, 6, and 10 min to identify the most suitable flight test duration. For each combination, five biological replicates were conducted, with the Kruskal-Wallis test used for statistical comparison of escape rates across different durations. The optimal setup for evaluating flight ability was determined to be 100 males per Petri dish in a 90 cm high cylinder, with escape rates recorded over a 6-minute period.
